# Comment on: “Effects of resistance training intensity on muscle quantity/quality in middle‐aged and older people: a randomized controlled trial” by Otsuka et al.

**DOI:** 10.1002/jcsm.13036

**Published:** 2022-07-21

**Authors:** Tim Kambič, Jerneja Farkaš, Mitja Lainscak

**Affiliations:** ^1^ Cardiac Rehabilitation Unit, and Department of Research and Education General Hospital Murska Sobota Murska Sobota Slovenia; ^2^ National Institute of Public Health Ljubljana Slovenia; ^3^ Faculty of Medicine University of Ljubljana Ljubljana Slovenia; ^4^ Division of Cardiology General Hospital Murska Sobota Murska Sobota Slovenia

Resistance training (RT) is recommended to counteract the deleterious effects of sarcopenia on muscle mass and function.^1^, ^2^, ^3^ The evidence about optimal RT scheme (training intensity, volume, rest, and so on) for optimal muscle outcomes in older individuals with and without sarcopenia as assessed by novel and recently recommended measurement techniques (magnetic resonance imaging, computed tomography, bioimpedance, dual‐energy X‐ray absorptiometry)^1^, ^2^ remains limited.

We therefore read with great interest the recent paper in the journal by Otsuka and colleagues,^4^ which evaluated the effects of different RT protocols (low‐load (LL‐) RT and moderate load (ML‐) RT) versus control on muscle quantity, quality and strength in healthy older adults. After 24 weeks of the intervention, a significant increase in cross‐sectional area of lower limb muscles in RT groups, and a significant improvement in some bioimpedance parameters (phase angle and membrane capacitance) in ML‐RT group was demonstrated. While both RT groups were superior to control group in improvement of cross‐sectional area of lower limb muscle and phase angle at 12 and 24 weeks, there was no significant difference between RT groups in muscle quantity and quality.^4^ Despite novel findings on muscle mass and quality, the study RT protocol needs some attention. In contrast to recent RT guidelines in healthy older adults,^3^ the authors compared only the effects of LL‐RT [40% of one repetition maximum (1‐RM)] and ML‐RT (60% of 1‐RM)^4^; both of them may present a suboptimal stimulus to induce muscle hypertrophy and strength gains.^3^, ^5^ Current recommendations are also supported by the previous two meta‐analysis showing the safety and superiority of high‐load (HL‐RT) over ML‐RT and LL‐RT on muscle strength, and similar effects on muscle hypertrophy in healthy young and older adults.^6^, ^7^ Furthermore, the cumulative training load should have been balanced between LL‐RT and ML‐RT groups by a number of repetitions (both RT groups performed three sets of 14 repetitions). In addition, the repetition range at a given RT load was suboptimal, as RT recommendations suggest the use of more than 15 repetitions/set when exercising at the intensities lower than 65% of 1‐RM.^8^

We believe that the study protocol as designed was suboptimal for potential muscle outcomes and may largely explain the lack of difference between RT groups for most of muscle hypertrophy measures as well as maximal muscle strength. What the authors do not report but it would be interesting to know is whether there was a difference in improvement of 1‐RM between each RT group and control group, similar as they reported for muscle quality and quantity outcomes.

As the sarcopenia trajectory severely affects muscle mass and function, it is important to use optimal RT to counteract such changes. The excellent study by Otsuka *et al*.^4^ has demonstrated novel insights in the dose‐dependent relationship between RT load and changes in muscle hypertrophy and strength. While there is a mounting evidence that muscle atrophy can be attenuated using variety of RT intensities (from LL to HL),^6^, ^7^ clinicians should also be aware on its negative impact of muscle function. With ageing, the loss of muscle mass is closely related to muscle denervation and a decrease in circulating anabolic hormones (e.g. growth hormone, insulin‐like growth factors I and II),^1^, ^9^, ^10^ which may be counteracted by the use of HL‐RT, especially given its superiority over LL‐RT in improvement of maximal muscle strength and activation.^3^, ^6^, ^7^ Additionally, the recent studies and guidelines in chronic disease patients (such as cancer,^11^, ^12^ coronary artery disease,^13^ chronic kidney disease,^14^, ^15^, ^16^ chronic pulmonary disease^17^) with higher risk of sarcopenia^2^ have replaced LL‐RT with progressive ML‐to‐HL‐RT or solely HL‐RT. Currently, cumulating evidence demonstrates beneficial effects of ML‐RT and HL‐RT on muscle hypertrophy and muscle strength compared with standard care or even with aerobic training alone.

We therefore are proponents to include HL‐RT as a core component in primary and secondary prevention of sarcopenia in older adults and patients. Further research should primarily focus on the safety, feasibility and efficacy of HL‐RT compared with other RT modalities in terms of maximal muscle strength and activation, anabolic hormone signalling pathways, and muscle quality and quantity in sarcopenic and/or cachectic patients.
